# Can you detect early dementia from an email? A proof of principle study of daily computer use to detect cognitive and functional decline

**DOI:** 10.1002/gps.4863

**Published:** 2018-02-09

**Authors:** G. Stringer, S. Couth, L.J.E. Brown, D. Montaldi, A. Gledson, J. Mellor, A. Sutcliffe, P. Sawyer, J. Keane, C. Bull, X. Zeng, P. Rayson, I. Leroi

**Affiliations:** ^1^ Faculty of Biology, Medicine and Health The University of Manchester Manchester UK; ^2^ School of Computer Science The University of Manchester Manchester UK; ^3^ Computing and Communications Lancaster University Lancaster UK; ^4^ Computer Science, School of Engineering and Applied Science Aston University Birmingham UK

**Keywords:** Alzheimer's disease, cognitive decline, computer use, dementia, functional ability, mild cognitive impairment

## Abstract

**Objective:**

To determine whether multiple computer use behaviours can distinguish between cognitively healthy older adults and those in the early stages of cognitive decline, and to investigate whether these behaviours are associated with cognitive and functional ability.

**Methods:**

Older adults with cognitive impairment (n = 20) and healthy controls (n = 24) completed assessments of cognitive and functional abilities and a series of semi‐directed computer tasks. Computer use behaviours were captured passively using bespoke software.

**Results:**

The profile of computer use behaviours was significantly different in cognitively impaired compared with cognitively healthy control participants including more frequent pauses, slower typing, and a higher proportion of mouse clicks. These behaviours were significantly associated with performance on cognitive and functional assessments, in particular, those related to memory.

**Conclusion:**

Unobtrusively capturing computer use behaviours offers the potential for early detection of neurodegeneration in non‐clinical settings, which could enable timely interventions to ultimately improve long‐term outcomes.

**Key points**

- This is one of the first investigations to explore a link between combined computer use behaviours and paper‐based instrumental activities of daily living.
- A profile of computer‐use behaviours can be used to differentiate between older adults with cognitive impairment and cognitively healthy older adults.
- Unobtrusively capturing data about various personal computer use behaviours could in the future be used to detect subtle, yet significant changes in cognitive and functional abilities.

**ABBREVIATIONS**
ACE: Addenbrooke's cognitive examination
AD: Alzheimer's disease
ECog: Measurement of Everyday Cognition
IADL: Instrumental activities of daily living
MCI: Mild cognitive impairment

## 1 INTRODUCTION

Impairments in cognitive and functional abilities can be detected in the prodromal or “mild cognitive impairment (MCI)” stage of dementia.^1^ Identifying the earliest symptoms of MCI is important for predicting progression to dementia and in providing a target for potential therapeutic interventions which act in the earlist stages of neurodegenerative diseases such as Alzheimer disease (AD).^2^ Current clinical diagnostic criteria for MCI include problems in performing instrumental activities of daily living (IADL) as a part of the clinical syndrome.^1^ IADL are activities beyond basic self‐care that are necessary for living independently, eg, cooking.^3^ Subtle impairments in IADL may also be evident in the pre‐clinical (ie, pre‐MCI) stage of dementia^4^, ^5^ and may be predictive of future cognitive decline.^6^ Moreover, higher‐level IADL, such as driving, managing finances, and using a computer, require complex cognitive processing and therefore may be more prone to deterioration in the early course of cognitive decline.^5^

To date, IADL assessments have generally been paper‐based tools which are intermittently administered in clinic settings, and which rely on the recall of past behaviour, either by the affected person or their informant. Such tools are not ideally suited to detecting subtle changes in an individual's functional ability in everyday settings, over a prolonged period of time.^7^, ^8^ The challenge, therefore, is to detect objective and meaningful functional changes in higher‐level IADL as early as possible and in ecologically meaningful settings, such as in the person's own home.

Capturing information about daily personal computer use activities may provide an opportunity to assess subtle changes in functional ability in elderly people over time. While personal computer use is an IADL in its own right, it also enables the user to complete a range of other complex IADLs, such as shopping, managing finances, and communicating.^9^ The number of adults aged over 65 years using technology in the UK is increasing. Daily computer use in this age‐group rose from 9% in 2006 to 45% in 2015,^10^ accessing the internet on a mobile phone grew from 3% in 2011 to 21% in 2016,^11^ and shopping online increased from 16% in 2008 to 45% in 2016.^11^ Furthermore, as competent computer use relies on intact cognitive functioning across several domains (eg, attention, working memory, and executive function), changes in patterns of computer use (ie, functional change) may be a particularly sensitive indicator of cognitive decline.^12^

Previous studies have demonstrated the feasibility of measuring computer use behaviours in older adults to distinguish between those with and without cognitive impairment. For example, it has been shown that people with MCI have reduced frequency and duration of daily computer use,^13^ and take longer to complete an online questionnaire.^14^ Seelye and colleagues^7^ have also demonstrated that people with MCI make significantly fewer mouse movements, take longer pauses between movements, and have a higher variability in the trajectory of mouse movements. These behaviours were significantly correlated with cognitive test scores. Vizer and Sears^15^ also demonstrated that keystroke speed and linguistic content is associated with cognitive impairment in older adults. In spite of these promising findings, it remains uncertain whether these individual computer use behaviours (eg, speed of use, typing abilities, and mouse operations) could be used as a composite marker of cognitive impairment in a single participant group. This is particularly important because a range of different behaviours are required to correctly operate a computer, and any one of these could be affected by cognitive decline. Another uncertainty in the field arises from the inclusion of novice or non‐computer users in the participant sample of previous studies (eg, Kaye et al^13^), which may limit the interpretation of findings due to the additional cognitive burden of learning to use a computer for the purposes of the study. Finally, the relationship between functional ability reflected by personal computer use and paper‐based IADL measures has yet to be explored.

The study presented here is a cross‐sectional proof of principle study designed to determine (1) whether multiple computer use behaviours, displayed by a sample of experienced older computer users on commonly undertaken computer tasks, can be used to distinguish between cognitively healthy older adults and those in the early stages of cognitive decline; and (2) whether these computer use behaviours are associated with cognitive and functional ability.

## 2 METHODS

### 2.1 Participants

Twenty participants with cognitive impairment (MCI, *n* = 17; mild dementia due to AD, *n* = 3) were recruited through the UK dementia research registry “Join Dementia Research”, as well as through local memory clinics and community groups. Participants referred from memory clinics had all received a clinical diagnosis from a qualified memory specialist based on Peterson's criteria^16^ for MCI or NINCDS‐ADRDA criteria^17^ for AD. Participants who self‐referred to the study all reported a diagnosis of MCI or mild dementia due to AD, given by a specialist memory clinic. Specific clinical subtypes of MCI (ie, amnestic vs non‐amnestic; single vs multiple domain) could not be ascertained. All participants had high functional ability, according to Katz criteria (all ≥5).^18^

Twenty‐four healthy control participants who had no prior history of cognitive impairment also participated in the study and were recruited through Join Dementia Research and local community groups (see Table 1 for demographic details).

**Table 1:** Demographic, computer use, cognitive, and functional variables

	Cognitively Healthy Control Participants, Mean (SD) *n* = 24	Cognitively Impaired Participants, Mean (SD) *n* = 20	Test Statistic	df	*P* value
Age (years)	71.09 (5.38)	75.60 (5.78)	−2.67	42	.011
Gender (% women)	58	30	3.532	1	.060^a^
Years of formal education	14.42 (3.88)	12.80 (3.74)	1.40	42	.169
15+ years computer use experience	19 (79.2%)	9 (45%)			.058^b^
Uses computer everyday	21 (87.5%)	11 (55%)			.015^b^
Trails B	81.17 (19.95)	145.45 (73.55)	−4.26	28.41^d^	.000
ACE‐III^c^ Total score	93.29 (4.05)	85.35 (6.92)	4.74	42	.000
ACE‐III^c^ Memory	23.96 (2.37)	20.30 (3.64)	3.86	31.51^d^	.001
ACE‐III^c^ Attention	17.42 (1.02)	16.6 (2.23)	1.51	25.51^d^	.116
ACE‐III^c^ Fluency	11.0 (1.84)	9.40 (2.04)	2.74	42	.009
ACE‐III^c^ Language	25.58 (.78)	24.50 (1.19)	3.49	31.53	.001
ACE‐III^c^ Visuospatial	15.33 (.91)	14.55 (1.79)	1.77	27.14	.088
ECog^e^ Total score	1.40 (.38)	2.06 (.72)	−3.75	27.72^d^	.001
ECog^e^ Memory	1.74 (.52)	2.71 (.81)	−4.66	31.12^d^	.000
ECog^e^ Language score	1.45 (.46)	2.11 (.90)	−2.97	27.18^d^	.006
ECog^e^ Visual‐spatial	1.24 (.37)	1.58 (.644)	−2.20	42	.003
ECog^e^: Planning	1.23 (.46)	1.92 (1.03)	−2.75	25.37^d^	.011
ECog^e^ Organization	1.20 (.37)	1.69 (.80)	−2.48	25.68^d^	.020
ECog^e^ Divided attention	1.38 (.63)	2.29 (.99)	−3.58	31.06	.001

a Chi square test.

b Mann Whitney test.

c ACE‐III, Addenbrooke's Cognitive Examination‐III.

d Equal variances not assumed.

e ECog, Measurement of Everyday Cognitive Function.

Bonferroni corrected *P* value (α = .003).

Additionally, to be included in the study, all participants were required to have the capacity to provide informed consent, were 65 years of age or older, were regular computer users (defined as using a laptop or desktop computer at least once a week), used Microsoft Windows versions 7, 8, or 10, were able to communicate verbally in English, and had no acute physical or mental problems severe enough to interfere with the conduct of the study.

Duration (in years) and current frequency (days per week) of computer use was recorded for each participant as a measure of computer use experience (Table 1).

The study was approved by the Health Research Authority―National Research Ethics Service England in accordance with the Declaration of Helsinki, and all participants provided informed consent to participate.

### 2.2 Procedure

Participants were invited to take part in a single testing session lasting approximately 2 hours conducted either in their own homes or at The University of Manchester.

#### 2.2.1 Cognitive and functional measures

Descriptive measures of global cognitive status were obtained using the Addenbrooke's Cognitive Examination (ACE)‐III.^19^ This test assesses 5 cognitive subdomains: attention, memory, verbal fluency, language, and visuospatial abilities, which provide a cognitive score out of a maximum of 100. Given that the only performance‐based measure of executive function on the ACE‐III is verbal fluency, we also incuded Part B of the Trail Making Test in the test battery as a measure of visual attention and task switching abilities.^20^

Subjective ratings of cognitive and functional capacity were obtained using the Everyday Cognition (ECog) scale.^21^ This assessment requires participants to rate their current functional abilities compared with 10 years previously. The 39‐item questionnaire assesses cognitively based functional items, across 6 domains: memory, language, visuospatial abilities, planning (executive functioning), organisation (executive functioning), and divided attention (executive functioning). Scores range from 1 (“Better or no change”) to 4 (“Consistently much worse”). To ensure high accuracy and detail of ECog ratings for cognitively impaired individuals, this test was completed by an informant (for 17 of the 20 participants) who knew the participant well, either as co‐habitants or seeing the participant in‐person at least 3 times per week.

Each group's mean total ACE‐III and ECog scores and mean scores for each cognitive domain (including Trail Making Test Part B) can be seen in Table 1.

#### 2.2.2 Tasks of computer performance

All tasks assessing computer use performance were completed on a laptop (Lenovo Think Pad T540P) running Windows 7, 8, or 10, depending on which operating system the participant was familiar with from their own personal computer. Participants were provided with a separate keyboard and mouse if they preferred.

Participants were asked to follow a set of written instructions in order to complete 4 experimental computer tasks: (1) a basic Desktop navigation task, which included using the date and time function, use of folders, and the recycle bin; (2) a Word processing task that involved editing a Word document and writing a diary entry; (3) an email (Outlook) task that included opening, writing, sending, and deleting emails; and (4) an internet browsing (Internet Explorer) task that included performing a Google search and navigation of a webpage. Participants could follow the instructions verbatim or adopt their own methods to complete the tasks, if they preferred.

Participants initially completed a practice session that involved shorter versions of the experimental computer tasks. The practice activity was repeated until the participant was confident in completing the tasks (approximately 2 repeats).

#### 2.2.3 Computer use behaviour data capture

Specially developed recording software (for further details, see Gledson et al^22^ and Bull et al^23^) captured computer use behaviours as a list of time‐stamped events. In‐line with previous research, the current study focussed on behaviours relating to mouse operations,^7^ keystrokes,^15^ and speed of use.^13^

Pauses were recorded as any period of inactivity greater than 10 seconds. To calculate event frequencies (eg, number of pauses per minute), computer use variables were divided by the total time to complete all 4 computer activities. Keyboard presses, and the key type and duration were recorded. Keystrokes included text‐based entries whilst completing the diary entry during the Microsoft Word task (based on Vizer and Sears^15^), as well as all other key‐presses for general computer operations. To distinguish keyboard presses relating to higher‐level linguistic and semantic features from more general operations, we analysed these separately and termed these “Text” and “Operational” keystrokes, respectively. Mouse operations included information such as total mouse clicks and the time, distance, and screen areas crossed.

### 2.3 Statistical analysis

Outliers for each computer use variable were removed using the non‐recursive procedure^24^ for each group of participants. This equated to 3.5% and 4.5% of data removed for the cognitively healthy control and the cognitively impaired groups, respectively. The distribution of the data was assessed using skewness and kurtosis. For non‐normally distributed variables, the data were log transformed.

Cross‐sectional group comparisons of demographic details, cognitive and functional test scores, and computer use variables were undertaken using independent samples t‐tests for continuous variables, Chi‐square tests for categorical variables, and Mann‐Whitney U tests for ordinal data. Kendall's Tau correlations were used to examine the relationship between selected computer use variables and each of the cognitive domains and total scores from the cognitive and functional paper‐based tests. To determine whether age and computer use experience could account for any associations observed between ECog and ACE‐III scores and selected computer use variables, separate hierarchical regression analyses were conducted for each of the computer use variables. In step one of each model, years and frequency of computer use were added to the regression. In step two, age was added. In step three, ECog and ACE‐III scores were added.

The selected computer use variables and the cognitive and functional test scores were then used to determine their probability distribution with respect to their sensitivity and specificity at classifying cognitive impairment using receiver operating characteristic (ROC) curve analyses. Predictive probability scores were calculated for the combined computer use variables and for the combined ACE‐III, ECog, and Trail Making Test B scores, and then also subject to ROC curve analyses. Comparisons between ROC curves were conducted according to the method described by DeLong et al.^25^

Analyses were performed using SPSS version 22 and MedCalc version 17.8.

## 3 RESULTS

### 3.1 Selection of candidate variables: Performance on computer tasks

Participants in the cognitively impaired group differed significantly from those in the control group on several computer use behaviours (Table 2).

**Table 2:** Comparison of selected computer use behaviours in cognitively healthy control participants compared with those with cognitive impairment, using independent samples t‐tests

		Cognitvely Healthy Control Participants			Cognitively Impaired Participants			*t* Value	df	*P* Value
		*N*	Mean	SD	*N*	Mean	SD			
Overall performance time	Total duration (min)	24	18.62	4.70	19	27.02	7.33	−4.56	41	<.001
	Total number of pauses	24	20.00	8.24	19	35.68	13.55	−4.69	41	<.001
	Number of pauses per min^a^	24	1.04	.24	19	1.35	.25	−4.08	41	<.001
	Pause length per pause	23	17.53	2.95	19	19.17	2.98	−1.78	40	.082
	Pause length per min	23	18.81	6.11	19	27.16	6.41	−4.32	40	<.001
Keyboard	Total “text” keystrokes	23	384.48	128.78	19	203.05	122.93	4.64	40	<.001
	“Text” keystrokes per min^a^	23	128.48	35.03	19	63.65	32.64	6.16	40	<.001
	Total “operational” keystrokes	23	122.26	20.98	18	133.11	31.31	−1.33	39	.192
	“Operational” keystrokes per min	23	8.30	2.14	18	5.43	1.86	4.50	39	<.001
Mouse	Total mouse clicks^a^	22	103.41	21.56	20	174.65	79.34	−3.88	21.55^b^	.001
	Mouse clicks per min	23	5.95	1.52	19	5.89	2.41	.095	29.21^b^	.925
	Inter‐click interval (secs)	23	10.7	2.49	20	11.3	4.43	−.595	29.0^b^	.557
	Total pixel count	24	21.5 k	8.77 k	17	22.2 k	9.32 k	−.253	39	.802
	Pixels per sec per inter‐click interval	23	20.1	5.78	20	15.2	6.39	2.60	41	.013

a Variables selected for further analysis.

b Equal variances not assumed.

Bonferroni corrected *P* value (α = .004).

#### 3.1.1 Overall performance time variables

Compared with participants in the control group, cognitively impaired participants took longer to complete the computer tasks, paused more frequently overall and per minute, and had a longer total pause length per minute. By contrast, the mean duration for each pause did not differ significantly between the 2 groups. Therefore, the number of pauses per minute was chosen as the focus of further analysis based on the assumption that the greater total pause length per minute for the cognitively impaired group is due to them taking more pauses (of similar duration to control participants) per minute.

#### 3.1.2 Keyboard‐use variables

Cognitively impaired participants made fewer “Text” keystrokes in total and per minute than the cognitively healthy participants. Because all participants took approximately the same length of time to complete the task involving “Text” keystrokes (approximately 3 minutes per participant), so total Text keystrokes and Text keystrokes per minute are a similar measure. Therefore, we focussed our analysis on Text Keystrokes per minute (ie, speed of typing). The cognitively impaired group did not differ significantly from the control group on total “Operational” keystrokes, but produced significantly fewer “Operational” keystrokes per minute. This difference was due to the different speeds the participants took to complete the tasks overall (see Section 3.1.1), and thus no further analysis was conducted on “Operational” keystrokes.

#### 3.1.3 Mouse‐based variables

The cognitively impaired group executed a significantly greater number of mouse clicks compared with the control group, but there were no group differences on the number of clicks per minute. We selected total mouse clicks for further analysis based on the assumption that this indicated cognitively impaired older adults made more mistakes and then had to perform more clicks to correct these errors and therefore also contributing to the longer total duration to complete the tasks (see Section 3.1.1). The time between clicks (ie, inter‐click interval) did not differ between the 2 groups. Mouse movements did not differ between the groups, as ascertained by the total number of pixels (ie, screen area covered) and the screen pixels within inter‐click intervals (ie, speed of mouse movements).

### 3.2 Correlations between computer use variables

Separate Kendall's Tau correlation analyses were conducted between the computer use variables selected from the group comparisons and each of the cognitive (ACE‐III and Trail Making Test Part B; Table 3) and functional (ECog; Table 4) measures. A number of significant correlations were found (all *P* < .05), but only the Memory domain of the ACE‐III and the ECog tests were significantly correlated with all 3 of the computer use variables.

**Table 3:** Correlation matrix for Trails B, ACE‐III, and computer use variables

Measure	1	2	3	4	5	6	7	8	9	10
1. Trails B	‐									
2. ACE Total	−.425^c^	‐								
3. ACE Attention	−.257^a^	.447^c^	‐							
4. ACE Memory	−.234^a^	.694^c^	.319^a^	‐						
5. ACE Fluency	−.370^b^	.481^c^	.118	.258^a^	‐					
6. ACE Language	−.326^b^	.559^c^	.236	.522^c^	.223	‐				
7. ACE Visuospatial	−.354^b^	.390^b^	.416^b^	.167	.149	.168	‐			
8. Number of pauses per min	.331^b^	−.376^c^	−.110	−.362^b^	−.298^b^	−.248^a^	−.154	‐		
9. “Text” keystrokes per min	−.474^c^	.519^c^	.153	.384^c^	.428^c^	.271^a^	.310^a^	−.358^c^	‐	
10. Total mouse clicks	.211	−.213	−.088	−.251^a^	−.148	−.198	−.024	.070	−.296^b^	‐

a *P* < .05.

b *P* < .01.

c *P* < .001.

**Table 4:** Correlation matrix for ECog and computer use variables

Measure	1	2	3	4	5	6	7	8	9	10
1. ECog Total	‐									
2. ECog Memory	.791^c^	‐								
3. ECog Language	.692^c^	.517^c^	‐							
4. ECog Visual–spatial	.722^c^	.624^c^	.612^c^	‐						
5. ECog Planning	.671^c^	.586^c^	.520^c^	.640^c^	‐					
6. ECog Organization	.582^c^	.476^c^	.444^c^	.512^c^	.540^c^	‐				
7. ECog Divided attention	.673^c^	.599^c^	.499^c^	.542^c^	.594^c^	.522^c^	‐			
8. Number of pauses per min	.175	.269^a^	.095	.094	.072	.184	.097	‐		
9. “Text” keystrokes per min	−.134	−.251^a^	−.051	−.121	−.081	−.120	−.128	−.358^e^	‐	
10. Total mouse clicks	.317^b^	.360^c^	.179	.158	.202	.208	.347^b^	.070	−.296^b^	‐

a *P* < .05.

b *P* < .01.

c *P* < .001.

Given that only the Memory domains were significantly correlated with all 3 computer use behaviours, we only included this cognitive domain within the regression models (Table 5). For mouse clicks and pauses per minute, neither age nor computer use experience could account for performance on these measures (all *P* > .05); however, the addition of ACE‐III and ECog Memory scores led to a significant increase in the explained variance (*R* ^*2*^ change values both *P* < .05), and this model showed significant predictions of number of pauses per minute and number of mouse clicks (both *P* < .05). For “Text” keystrokes per minute, computer use experience was a significant predictor of performance accounting for 36.8% of the variability, which increased significantly with the addition of age (*R* ^*2*^ change = .101, *P* = .011) and increased significantly again with the addition of ACE‐III and ECog Memory scores (*R* ^*2*^ change = .103, *P* = .020). Therefore, ACE‐III and ECog Memory scores are significant predictors of keyboard typing speed (*R* ^*2*^ = .260, *P* = .003), but age and computer use experience may also account for variability in this behaviour.

**Table 5:** Hierarchical linear regression analysis to account for age and computer use experience in the variability of computer use performance

Dependent Variable	Model	*R* Square	*F*	*P*	Change Statistics		
					*R* Square Change	*F* Change	*P*
Number of pauses per min	Step 1	0.045	0.948	0.396	‐	‐	‐
	Step 2	0.152	2.321	0.090	0.106	4.885	0.033*
	Step 3	0.356	4.089	0.005*	0.204	5.870	0.006*
“Text” keystrokes per min	Step 1	0.368	11.430	<.001*	‐	‐	‐
	Step 2	0.469	11.179	<.001*	0.101	7.233	.011*
	Step 3	0.572	9.629	<.001*	0.103	4.348	.020*
Total mouse clicks	Step 1	0.124	2.767	0.075	‐	‐	‐
	Step 2	0.130	1.901	0.146	0.006	0.271	0.605
	Step 3	0.319	3.379	0.013*	0.189	4.997	.012*

Step 1, years and frequency of computer use; Step 2, + age; Step 3, + ACE‐III and ECog Memory scores.

* *P* < .05

### 3.3 Accounting for within‐group differences

To account for the possibility that the between‐group differences were driven by those with mild dementia due to AD, all statistical analyses were repeated comparing only MCI participants to control participants. The results were unaffected, with the exception of ACE‐III Memory score, which was no longer significantly related to number of mouse clicks.

### 3.4 Receiver operating characteristic curve (ROC) analysis

The ROC analyses (Table 6) for the computer use variables all showed “good” (AUC = .8–.9) or “excellent” (AUC = .9–1.0) correct classification of cognitive impairment. In comparison, ACE‐III and ECog total scores and memory domain scores, as well Trail Making Test B scores, all showed “moderate” (AUC = .7–.8) or “good” correct classification of cognitive impairment. Sensitivity and specificity values for each measure, as determined from the Youden index (*J*), are included in Table 6. When all the selected computer use variables were combined into a single predictive probability and compared with combined ACE‐III Memory score, ECog Memory score, and Trail Making Test B predictive probability, correct classification was significantly higher for the combined computer use variables (*z* = 2.002, *P* = .045).

**Table 6:** ROC curve analyses

Variable	Area under the ROC Curve			Youden Index				
	AUC	SE	*z*	*P*	*J*	Cut‐off criteria	Sensitivity (%)	Specificity (%)
Number of pauses per min	0.80	0.07	4.47	<.001	0.509	>1.09	84.21	66.67
“Text” keystrokes per min	0.91	0.04	9.98	<.001	0.677	≤104	89.47	78.26
Total mouse clicks	0.80	0.08	3.86	<.001	0.550	>146	55.00	100.00
ACE‐III Total	0.85	0.06	5.62	<.001	0.675	≤89	80.00	87.50
ACE‐III Memory	0.80	0.07	4.47	<.001	0.558	≤23	85.00	70.83
ECog Total	0.82	0.07	4.74	<.001	0.575	>1.26	95.00	62.50
ECog Memory	0.84	0.06	5.26	<.001	0.600	>2.00	85.00	75.00
Trail making test B (seconds)	0.83	0.06	5.35	<.001	0.525	>82	90.00	62.50
Computer use behaviours combined	0.98	0.02	26.13	<.001	0.889	>.72	88.89	100.00
Memory (ACE‐III and ECog) and trails B combined	0.92	0.04	9.89	<.001	0.717	>.41	80.00	91.67

## 4 DISCUSSION

In this proof of principle study, we examined whether computer use behaviours recorded from semi‐structured tasks could discriminate between people with cognitive impairment and cognitively healthy control participants, and whether measures of functional ability and cognition were related to these computer use behaviours. Consistent with previous findings, the 2 groups performed differently on computer activity measures of time,^13^ keystrokes,^15^ and mouse operations.^7^ In contrast to previous studies which have focused on individual examples of computer use behaviour, here we have demonstrated that a *combined* profile of behaviours has potential to provide information about cognitive and functional decline in the early stages of neurodegeneration. We have also demonstrated the potential influence that age and computer use experience can have on computer use abilities and therefore need to be accounted for when determining how cognitive ability affects computer use performance.

Decline in performance of computer‐based activities is likely to vary among individuals; therefore, capturing a range of behaviours will significantly increase the likelihood of early detection. Nonetheless, when capturing data reflecting multiple behaviours, it is imperative that the measures are highly sensitive and specific to acknowledged thresholds for recognised clinical syndromes such as MCI or dementia, thus guarding against a high false positive rate. In the current study, all of the computer use measures showed “good” or “excellent” correct classification of cognitive impairment with high sensitivity and specificity. Indeed, when these measures were combined into a single predictive probability measure, they showed a significantly greater correct classification of cognitive impairment compared with a combination of paper‐based measures typically used in a clinical setting. Additionally, certain participants within the cognitively impaired group scored within the normal range on the ACE‐III (>88/100), which could explain why the specificity and sensitivity of ACE‐III scores were lower than reported previously (ie, a cut‐off score of <88 giving 100% sensitivity and 96% specificity^19^). This could be due to numerous reasons, including a practice effect from completing the test previously in clinic, the home setting being a less stressful environment compared with a clinic setting, and/or the day‐to‐day variability in cognitive functioning as a result of changes in mood or fatigue. Therefore, this emphasises the utility of these computer‐based monitoring measures to provide a potentially sensitive identification of cognitive impairment in a home‐based setting in the first instance, which could then be used to supplement follow‐up clinic‐based measures to ascertain the degree and type of impairment.

One limitation of this exploratory study is that sub‐type of MCI (ie, amnestic vs non‐amnsetic) of each participant was unknown. We acknowledge, therefore, that there may have been some variability in cognitive profiles between participants. From the ACE‐III and ECog results, there are clear group differences on numerous cognitive domains, but only memory scores were significantly correlated with all 3 of the selected computer use variables. It remains unclear why episodic and semantic memory abilities (which are included in the ACE‐III and ECog tests) may be related to such functional tasks as keyboard typing speed. It could be that the majority of participants were of amnestic MCI type, and so memory was the strongest measure of overall cognitive function (as assessed by the ACE‐III). Similarly, because the ECog was completed mostly by participants' informants, perhaps memory decline is the most noticeable impairment compared with other cognitive domains and is therefore rated as the most impaired domain. To address this issue, it would be beneficial to use a cognitive test battery which covers a broader range of cognitive domains, such as procedural memory and processing speed. Nevertheless, it remains uncertain which computer use behaviour changes (eg, slower typing speed) are most likely to be associated with declines in particular cognitive functions (eg, divided attention, language production, procedural memory, etc.). We have recently attempted to address this issue by convening a group of experts in clinical and cognitive neuroscience to determine which cognitive domains may be related to a range of different computer use behaviours, and how decline in specific domains might affect performance on different computer use activities (see Couth et al^26^).

## 5 CONCLUSION

This proof of principle study has demonstrated that a computer‐based monitoring system can differentiate between cognitive impairment (ie, MCI and early AD) and healthy cognitive ageing using semi‐directed computer tasks and several objective measures of computer use performance. The next phase will be to determine whether we can passively detect early changes over time in these same computer use behaviours, using unobtrusive recording of the behaviours through software embedded in participants' personal computers. The ultimate aim is to ascertain whether behaviour changes associated with cognitive and functional decline could provide a sensitive and efficient way to detect very early signs of dementia.

## CONFLICT OF INTEREST

None declared.
